# Predictors of Pacemaker Implantation Among Patients Hospitalized With Syncope: An Analysis of the National Inpatient Sample (2016-2020)

**DOI:** 10.7759/cureus.107897

**Published:** 2026-04-28

**Authors:** Emmanuel Kokori, Samuel Osei, Nirajan Kandel, Zuhaib Khokar, Abdullah Ahmad, Muhammad Daniyal, Syed Hasham Ali

**Affiliations:** 1 Department of Internal Medicine, Cape Fear Valley Health, Fayetteville, USA; 2 Department of Internal Medicine, Hameed Latif Teaching Hospital, Lahore, PAK; 3 Department of Internal Medicine, Montefiore Medical Center, Bronx, USA; 4 Department of Internal Medicine, Dow Medical College, Karachi, PAK

**Keywords:** bradyarrhythmia, cardiac conduction defects, national inpatient sample (nis), pacemaker, syncope

## Abstract

Introduction: Syncope is a frequent cause of hospitalization with etiologies ranging from benign to life-threatening. Identifying which patients will ultimately require permanent pacemaker implantation (PPI) remains a clinical challenge. We aimed to characterize the independent predictors of PPI using a nationally representative inpatient dataset to optimize clinical risk stratification and guide targeted diagnostic workups.

Methods: We analyzed the National Inpatient Sample (NIS) from 2016 to 2020, identifying adult patients hospitalized with a primary diagnosis of syncope (ICD-10-CM: R55). PPI was identified using ICD-10-PCS codes (0JH604Z, 0JH606Z, 0JH605Z). Univariate and multivariate logistic regression were performed to identify independent predictors of PPI, adjusting for patient demographics, hospital characteristics, and clinical comorbidities. Survey weights were applied throughout to generate nationally representative estimates.

Results: Of 2,046,185 weighted syncope hospitalizations, 50,060 (2.4%) underwent PPI. Conduction disorders were the strongest independent predictor (adjusted odds ratio (aOR) 14.82; 95% CI 14.53-15.11), followed by age ≥65 years (aOR 4.10), atrial fibrillation (aOR 1.78), obesity (aOR 1.17), and hypertension (aOR 1.10). Factors such as Black race, Hispanic ethnicity, and Medicaid or self-pay status were associated with lower odds of PPI. Additionally, comorbid sepsis, malignancy, electrolyte imbalance, history of prior stroke, anemia, and chronic pulmonary disease were also independently associated with lower odds of PPI. Notably, diabetes mellitus (DM), congestive heart failure (CHF), chronic kidney disease (CKD), and coronary artery disease (CAD), each positive on univariate analysis, became significant negative predictors after multivariate adjustment.

Conclusions: Conduction abnormalities and older age above 65 years remain the dominant drivers of PPI in hospitalized syncope patients. The reversal of several common comorbidities to negative predictors after adjustment suggests they signal non-bradycardic etiologies and should prompt a more comprehensive evaluation before pacing is pursued. Significant racial and socioeconomic disparities in PPI highlight the need for targeted quality and equity initiatives.

## Introduction

Syncope accounts for approximately 1-3% of all emergency department visits and hospital admissions worldwide, presenting a significant diagnostic challenge and an economic burden on healthcare systems [1,2]. While the majority of syncope cases are benign or neurocardiogenic in nature, cardiac syncope, particularly that caused by symptomatic bradyarrhythmias or high-grade conduction system disease, is associated with increased morbidity and sudden cardiac death [3,4]. For these patients, timely identification of the need for permanent pacemaker (PPM) implantation is essential to prevent recurrent falls, injury, and mortality [5,6].

Despite established guidelines from the American College of Cardiology (ACC) and the European Society of Cardiology (ESC), predicting which patients hospitalized for syncope will ultimately require a PPM remains difficult in the acute setting [5,6]. Existing evidence largely derives from small, single-center, or highly selected cohorts, providing limited insight into real-world predictors. Consequently, a specific knowledge gap remains regarding how complex comorbidities and non-clinical factors, including socioeconomic and racial disparities, interact to influence access to pacing therapy. This narrow focus often fails to capture the complexity of the broader patient population encountered in daily practice [7].

There is a pressing need for a comprehensive, population-level analysis to refine the predictive profile of syncope patients at high risk for pacing interventions. This study addresses the exact unmet question of what independent factors drive real-world pacemaker implantation across the United States. The National Inpatient Sample (NIS), the largest publicly available inpatient healthcare database in the United States, provides a unique opportunity to evaluate these predictors across a diverse range of hospital types, geographic regions, and patient demographics [8]. By analyzing large-scale trends, we can better understand the clinical and socioeconomic determinants that drive the need for definitive pacing therapy in patients with syncope.

The objective of this study was to identify independent clinical, demographic, and hospital-level predictors of permanent pacemaker implantation (PPI) among adults hospitalized with syncope, utilizing a nationally representative sample from 2016 to 2020, to define the high-risk syncope population in the United States.

## Materials and methods

Data source

The study was conducted at Cape Fear Valley Health System, Fayetteville, NC, USA. This study utilized data from the NIS from 2016 to 2020 [8]. This timeframe was selected to ensure the exclusive use of ICD-10 coding, which was federally mandated in October 2015, providing uniform diagnostic and procedural classification throughout the study period. NIS is the largest publicly available healthcare database that accounts for a 20% stratified sample of discharges from U.S. community hospitals. Discharge weights provided by the Healthcare Cost and Utilization Project (HCUP) were applied to generate national estimates of hospitalizations. Because the NIS is a publicly available database containing fully de-identified patient information, this study did not meet the federal definition of human subjects research and was therefore deemed exempt from formal Institutional Review Board (IRB) review and informed consent requirements. Strengthening the Reporting of Observational Studies in Epidemiology (STROBE) guidelines were followed for this project.

Study population

Retrospective analysis of all adult (age >18 years) patients, hospitalized with the primary diagnosis of syncope, was performed. These patients were identified using the International Classification of Diseases, Tenth Revision, Clinical Modification (ICD-10-CM) code R55 [9]. Patients in whom syncope was coded solely as a secondary diagnosis, as well as those under 18 years of age, were excluded from the study cohort.

Variables and outcome measures

The primary outcome of interest was PPI, identified using ICD-10-Procedure Coding System (ICD-10-PCS) codes (0JH604Z, 0JH606Z, 0JH605Z).

The baseline characteristics included were demographics, including age (stratified into 18-44, 45-64, and ≥65 years); sex; race (White, Black, Hispanic, Asian/Pacific Islander, Native American, and Other individuals); and median household income quartile for the patient’s ZIP code. In the HCUP NIS database, the "Other" race category encompasses individuals whose race is not defined by the five primary groups (White, Black, Hispanic, Asian/Pacific Islander, and Native American individuals). This includes patients identifying with multiple races or those explicitly coded as "Other" by the reporting state or hospital. Hospital characteristics included the hospital region (Northeast, Midwest, South, West), teaching status/location, and admission type (elective vs. non-elective). Clinical comorbidities: To account for patient complexity, we adjusted for a range of covariates. It is important to note that these represent available administrative variables derived from billing codes, capturing documented comorbidity burden rather than the full granular clinical context driving real-world pacing decisions. Relevant comorbidities were identified using specific ICD-10-CM codes, including hypertension, diabetes mellitus (DM), atrial fibrillation, conduction disorders, congestive heart failure (CHF), coronary artery disease (CAD), malignancy, obesity, chronic kidney disease (CKD), chronic pulmonary disease, anemia, electrolyte imbalance, history of stroke/transient ischemic attack (TIA), and sepsis.

Statistical analysis

All statistical analyses accounted for the complex survey design of the NIS, including clusters, strata, and discharge weights, to produce nationally representative estimates. Baseline characteristics were compared between patients who received PPI and those who did not using Pearson’s chi-square test for categorical variables and independent t-tests for continuous variables.

Univariate logistic regression was performed to assess the unadjusted association between each predictor and PPI. Subsequently, a multivariate logistic regression model was constructed to identify independent predictors of PPI. Variables were selected for inclusion in the multivariable model based on established clinical relevance and statistical significance in the univariate analysis (defined a priori as P < 0.10). The final model adjusted for the aforementioned patient demographics, hospital-level characteristics, and clinical comorbidities.

Results are reported as odds ratios (OR) for univariate analysis and adjusted odds ratios (aOR) for multivariate analysis, with 95% confidence intervals (CI). A two-sided P-value of < 0.05 was considered statistically significant. Missing data for race and median household income, variables that were the most unavailable, were handled using complete-case analysis, consistent with prior NIS-based studies. Because the proportion of missing data for these specific demographic variables in the NIS is historically low (typically <3-5%), the complete-case approach was deemed appropriate without the need for multiple imputation techniques. All analyses were conducted using IBM SPSS Statistics for Windows, Version 23 (Released 2015; IBM Corp., Armonk, New York, United States).

## Results

Baseline characteristics

A total of 2,046,185 weighted hospitalizations for syncope were identified, of which 50,060 (2.4%) patients underwent PPI.

Patients in the PPI group were significantly older (82.8%, P < 0.001) and more likely to be male (52.3%, P < 0.001). Medicare beneficiaries accounted for the majority of pacemaker implantations (77.6%). This was a significantly higher proportion than in the non-PPI group (62.5%, P < 0.001). The PPI group was more likely to belong to the lower-income quartiles than to the higher-income quartiles. The PPI group had a higher proportion of White patients (80.9% vs. 68.4%) and a lower proportion of Black patients (7.0% vs. 16.0%) compared to the non-PPI group (P < 0.001).

Hospitals in the Midwest and urban teaching hospitals had the highest admissions in both groups (P < 0.001 for both comparisons). The majority of syncope admissions were non-elective; however, elective patients were more likely to belong to the PPI group (6.7 vs. 7.4%, P < 0.001).

Cardiac comorbidities were markedly higher in the PPI group. Around 60.6% of patients in the PPI group had conduction abnormalities compared to 8.6% in the non-PPI group (P < 0.001). Similarly, atrial fibrillation was more common in the PPI group (35.1% vs. 19.9%, P < 0.001). The PPI group also had higher rates of CHF, CAD, and CKD. DM was the only comorbidity that did not differ significantly between the two groups. Table 1 summarizes the prevalence of each baseline characteristic in both groups.

**Table 1:** Baseline demographic, clinical, and hospital characteristics of patients hospitalized with syncope, stratified by permanent pacemaker implantation status PPI: permanent pacemaker implantation; SD: standard deviation; TIA: transient ischemic attack; NIS: National Inpatient Sample Categorical variables are presented as n (unweighted) with weighted percentages. Continuous variables are presented as mean ± SD. P-values were derived using Pearson’s chi-square test for categorical variables and independent t-test for continuous variables, accounting for the complex survey design of the NIS

Variable	No Pacemaker (N = 1,996,125)	Pacemaker (N = 50,060)	Test Statistic	P-value
Age (Mean ± SD)	66.56 ± 18.34	75.18 ± 12.44	t = -150.85	<0.001
Age Group			—	<0.001
18–44 years	221,085 (11.2%)	1,145 (2.3%)		
45–64 years	525,070 (26.7%)	7,430 (14.9%)		
65+ years	1,219,840 (62.0%)	41,405 (82.8%)		
Sex			χ^2^= 294.95	<0.001
Male	967,225 (48.5%)	26,205 (52.3%)		
Female	1,028,575 (51.5%)	23,855 (47.7%)		
Race			χ^2^= 4001.70	<0.001
White	1,331,885 (68.4%)	39,295 (80.9%)		
Black	311,915 (16.0%)	3,410 (7.0%)		
Hispanic	182,720 (9.4%)	3,170 (6.5%)		
Asian/Pacific Islander	53,530 (2.8%)	1,320 (2.7%)		
Native American	8,485 (0.4%)	245 (0.5%)		
Other	57,775 (3.0%)	1,110 (2.3%)		
Median Household Income			χ^2^= 537.67	<0.001
Quartile 1 (Lowest)	586,520 (29.9%)	12,395 (25.1%)		
Quartile 2	502,950 (25.6%)	13,345 (27.0%)		
Quartile 3	463,985 (23.6%)	12,300 (24.9%)		
Quartile 4 (Highest)	408,540 (20.8%)	11,295 (22.9%)		
Primary Payer			χ^2^= 5641.31	<0.001
Medicare	1,245,520 (62.5%)	38,805 (77.6%)		
Medicaid	232,615 (11.7%)	1,960 (3.9%)		
Private Insurance	390,300 (19.6%)	7,610 (15.2%)		
Self-Pay	68,820 (3.5%)	625 (1.2%)		
Hospital Region			χ^2^= 71.41	<0.001
Northeast	430,040 (21.5%)	10,090 (20.2%)		
Midwest	787,690 (39.5%)	19,735 (39.4%)		
South	416,035 (20.8%)	10,920 (21.8%)		
West	362,360 (18.2%)	9,315 (18.6%)		
Admission Type			χ^2^= 37.40	<0.001
Non-elective (Emergency)	1,860,530 (93.3%)	46,310 (92.6%)		
Elective	132,770 (6.7%)	3,675 (7.4%)		
Comorbidities				
Hypertension	1,404,675 (70.4%)	40,110 (80.1%)	χ^2^= 2,246.2	<0.001
Conduction Disorders	171,420 (8.6%)	30,320 (60.6%)	χ^2^= 172,201.5	<0.001
Coronary Artery Disease	566,350 (28.4%)	18,200 (36.4%)	χ^2^= 1,507.4	<0.001
Atrial Fibrillation	397,220 (19.9%)	17,565 (35.1%)	χ^2^= 6,505.7	<0.001
Diabetes Mellitus	599,580 (30.0%)	15,140 (30.2%)	χ^2^= 0.99	0.320
Chronic Kidney Disease	405,885 (20.3%)	10,585 (21.1%)	χ^2^= 19.3	<0.001
Congestive Heart Failure	373,230 (18.7%)	9,750 (19.5%)	χ^2^= 21.2	<0.001
Obesity	265,670 (13.3%)	7,015 (14.0%)	χ^2^= 20.6	<0.001
History of Stroke/TIA	192,100 (9.6%)	3,665 (7.3%)	χ^2^= 307.3	<0.001
Sepsis	94,980 (4.8%)	510 (1.0%)	χ^2^= 1,854.6	<0.001

Patient-level and clinical predictors of permanent pacemaker implantation

In the univariate analysis, advanced age was strongly associated with increased odds of PPI among syncope patients. Compared to patients aged 18-44 years, those aged 45-64 years had nearly a three-fold increase in unadjusted odds, while patients aged ≥65 years had over a six-fold increase (P < 0.001 for both). Sex was also a significant predictor, with female patients demonstrating 14% lower unadjusted odds of receiving a pacemaker compared to males (P < 0.001).

Conduction disorders exhibited the strongest unadjusted association with pacemaker implantation, with greater than 16-fold higher odds (P < 0.001). Other significant positive predictors (P < 0.001 for all) included comorbid atrial fibrillation (two-fold increase), hypertension (70% increase), CAD (44% increase), obesity (6% increase), CKD (5% increase), and CHF (5% increase). Conversely, sepsis (79% lower odds), malignancy (51% lower), anemia (42% lower), electrolyte imbalances (40% lower), and history of stroke (26% lower) were associated with a reduced likelihood of implantation (P < 0.001).

After adjusting for confounders via multivariate logistic regression, conduction disorders remained the strongest independent predictor of PPI (aOR 14.82; P < 0.001). Age remained a robust predictor, with patients aged ≥65 years having four times the odds of receiving a pacemaker compared to those aged 18-44 years.

Atrial fibrillation (78% increase), obesity (17% increase), and hypertension (10% increase) remained significant independent positive predictors (P < 0.001). Conversely, sepsis (73% lower), malignancy (44% lower), electrolyte imbalance (38% lower), history of stroke (37% lower), anemia (29% lower), and chronic pulmonary disease (18% lower) continued to be independently associated with reduced odds of implantation (P < 0.001 for all).

Notably, after adjustment, sex was no longer a significant predictor (P = 0.119). DM, which was initially non-significant, emerged as a significant negative predictor in the adjusted model (5% lower odds; P < 0.001). Interestingly, CAD (5% lower), CKD (9% lower), and CHF (33% lower) all shifted to become negative predictors after adjusting for confounding variables.

Socioeconomic predictors

In the unadjusted analysis, racial minorities, including Black, Hispanic, Asian/Pacific Islander, and “Other” racial groups, were significantly less likely to receive a pacemaker compared to White patients (P < 0.001). Native American race was not a significant predictor in the univariate model (P = 0.740). However, after adjustment, Native American race was associated with 32% higher odds of receiving a pacemaker compared to White patients (P < 0.001), while Asian/Pacific Islander race was no longer significantly associated with decreased odds (P = 0.867). Black (49% lower), Hispanic (20% lower), and “Other” (18% lower) racial groups remained significant negative predictors (P < 0.001).

In terms of payer status, compared to Medicare, all other payer categories (Medicaid, private, self-pay, no charge, and other) had lower unadjusted odds of pacemaker implantation (P < 0.001). In the multivariate model, patients with private insurance had 16% higher odds of PPI compared to Medicare beneficiaries (P < 0.001), while the “Other” payer category became non-significant. Medicaid and self-pay status remained significant negative predictors (P < 0.001).

Socioeconomic trends regarding income also shifted after adjustment. In the unadjusted model, increasing income quartile was generally associated with higher odds of PPI (P < 0.001). In the adjusted model, while the second-lowest income quartile continued to have slightly higher odds (4% increase; P = 0.010) compared to the lowest quartile, the highest income quartile (P < 0.001) and second-highest quartile (P = 0.012) were associated with significantly lower odds of PPI.

**Figure 1:**
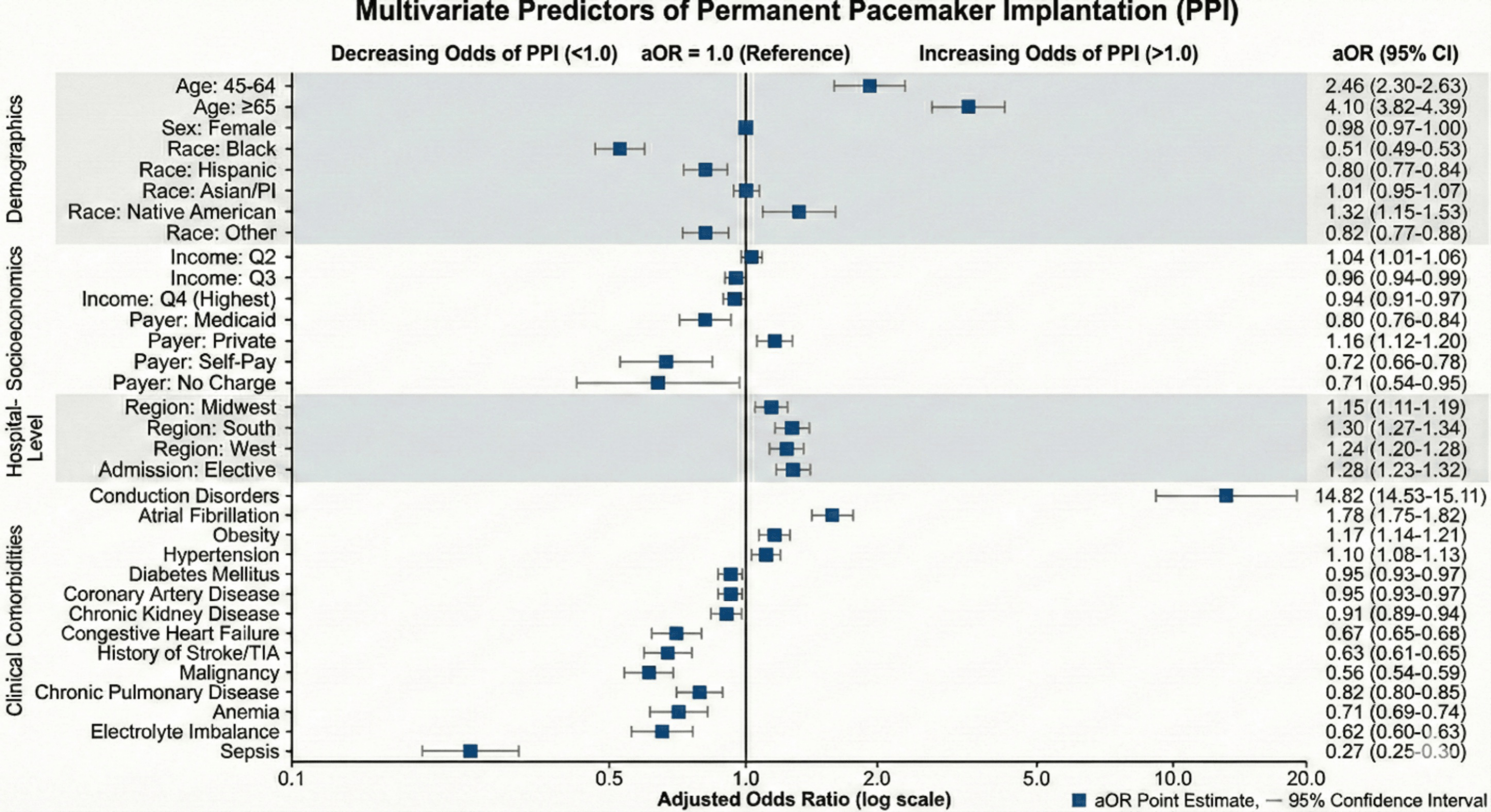
Forest plot of multivariate predictors of permanent pacemaker implantation (PPI) This forest plot illustrates the adjusted odds ratios (aORs) and 95% confidence intervals (CIs) for various predictors associated with PPI in patients hospitalized with syncope.

Regarding hospital characteristics, elective admission was associated with higher odds of PPI compared to non-elective admission in both unadjusted and adjusted models (P < 0.001). Geographically, hospitals in the Midwest, South, and West all demonstrated higher odds of PPI compared to the Northeast in both models (P < 0.001). Regarding hospital teaching status, it is important to note that the model utilized the 'unclassified/missing' status group as the statistical reference. Compared to this unclassified baseline, both rural (aOR 1.07; P = 0.002) and urban teaching hospitals (aOR 1.07; P = 0.001) demonstrated slightly higher odds of PPI (Table 2). However, because the reference group is unclassified, these odds ratios cannot be interpreted as direct pairwise comparisons between rural and urban facilities. Figure 1 is a forest plot of multivariate predictors of PPI.

**Table 2:** Univariate and multivariate logistic regression analysis identifying predictors of permanent pacemaker implantation in patients hospitalized with syncope aOR: adjusted odds ratio; CI: confidence interval; NIS: National Inpatient Sample; OR: odds ratio; PPI: permanent pacemaker implantation; TIA: transient ischemic attack Analyses accounted for the complex survey design of the NIS (clusters, strata, and discharge weights). *The reference group for hospital teaching status (n = 26,782) comprises cases with unclassified or missing teaching-status codes. ORs for rural, urban non-teaching, and urban teaching hospitals, therefore, reflect comparisons to this unclassified group, not pairwise comparisons to each other. Inferences regarding rural–urban disparities should be interpreted with caution. Model Goodness-of-Fit Statistics: Omnibus Test of Model Coefficients: χ2= 95,371.743; df = 37, p < 0.001. Nagelkerke R Square: 0.233.

Predictor	Univariate OR (95% CI)	Univariate Score χ2	P-Value	Multivariate aOR (95% CI)	Multivariate Wald χ2	P-value
Age Group (Ref: 18–44)		8950.89			539.99	
45–64 years	2.73 (2.57 – 2.91)	3315.95	<0.001	2.46 (2.30 – 2.63)	357.36	<0.001
<65 years	6.55 (6.18 – 6.95)	8475.36	<0.001	4.10 (3.82 – 4.39)	532.19	<0.001
Sex (Ref: Male)						
Female	0.86 (0.84 – 0.87)	274.81	<0.001	0.98 (0.97 – 1.00)	8.03	0.119
Race (Ref: White)		3821.97			1236.78	
Black	0.37 (0.36 – 0.38)	2815.03	<0.001	0.51 (0.49 – 0.53)	1148.84	<0.001
Hispanic	0.59 (0.57 – 0.61)	400.65	<0.001	0.80 (0.77 – 0.84)	104.39	<0.001
Asian / Pacific Islander	0.84 (0.79 – 0.88)	0.68	<0.001	1.01 (0.95 – 1.07)	0.01	0.867
Native American	0.98 (0.86 – 1.11)	67.85	0.740	1.32 (1.15 – 1.53)	16.12	<0.001
Other	0.65 (0.61 – 0.69)	2.93	<0.001	0.82 (0.77 – 0.88)	36.59	<0.001
Payer Status (Ref: Medicare)		4917.63			298.69	
Medicaid	0.27 (0.26 – 0.28)	2525.47	<0.001	0.80 (0.76 – 0.84)	50.23	<0.001
Private Insurance	0.63 (0.61 – 0.64)	484.99	<0.001	1.16 (1.12 – 1.20)	105.46	<0.001
Self-Pay	0.29 (0.27 – 0.32)	687.76	<0.001	0.72 (0.66 – 0.78)	49.45	<0.001
No Charge	0.25 (0.19 – 0.33)	69.69	<0.001	0.71 (0.54 – 0.95)	4.61	0.021
Income (Ref: Quartile 1 - Lowest)		539.16			45.33	
Quartile 2	1.26 (1.22 – 1.29)	42.71	<0.001	1.04 (1.01 – 1.06)	10.45	0.010
Quartile 3	1.25 (1.22 – 1.29)	38.96	<0.001	0.96 (0.94 – 0.99)	2.96	0.012
Quartile 4 (Highest)	1.31 (1.28 – 1.34)	145.48	<0.001	0.94 (0.91 – 0.97)	9.50	<0.001
Admission Type (Ref: Non-elective)						
Elective Admission	1.11 (1.08 – 1.15)	54.12	<0.001	1.28 (1.23 – 1.32)	195.46	<0.001
Hospital Region (Ref: Northeast)		70.19			330.35	
Midwest	1.12 (1.09 – 1.15)	11.98	<0.001	1.15 (1.11 – 1.19)	59.31	<0.001
South	1.07 (1.04 – 1.09)	0.06	<0.001	1.30 (1.27 – 1.34)	313.14	<0.001
West	1.10 (1.06 – 1.13)	18.60	<0.001	1.24 (1.20 – 1.28)	117.87	<0.001
Hospital Teaching Status*		897.98			656.39	
Rural	1.08 (1.04 – 1.12)	31.59	<0.001	1.07 (1.03 – 1.12)	168.70	0.002
Urban Non-Teaching	1.05 (1.01 – 1.10)	683.23	0.011	1.01 (0.96 – 1.05)	619.75	0.816
Urban Teaching	1.09 (1.05 – 1.13)	—	<0.001	1.07 (1.03 – 1.11)	—	0.001
Clinical Comorbidities						
Conduction Disorders	16.35 (16.06 – 16.63)	140484.63	<0.001	14.82 (14.53 – 15.11)	70636.06	<0.001
Atrial Fibrillation	2.17 (2.13 – 2.22)	6451.44	<0.001	1.78 (1.75 – 1.82)	2642.94	<0.001
Obesity	1.06 (1.04 – 1.09)	9.34	<0.001	1.17 (1.14 – 1.21)	164.07	<0.001
Hypertension	1.70 (1.66 – 1.73)	1715.54	<0.001	1.10 (1.08 – 1.13)	41.14	<0.001
Diabetes Mellitus	1.01 (0.99 – 1.03)	2.15	0.319	0.95 (0.93 – 0.97)	12.32	<0.001
Coronary Artery Disease	1.44 (1.42 – 1.47)	1268.27	<0.001	0.95 (0.93 – 0.97)	25.35	<0.001
Chronic Kidney Disease	1.05 (1.03 – 1.07)	7.37	<0.001	0.91 (0.89 – 0.94)	69.00	<0.001
Congestive Heart Failure	1.05 (1.03 – 1.07)	7.36	<0.001	0.67 (0.65 – 0.68)	957.45	<0.001
History of Stroke/TIA	0.74 (0.72 – 0.77)	300.10	<0.001	0.63 (0.61 – 0.65)	652.28	<0.001
Malignancy	0.49 (0.47 – 0.52)	901.04	<0.001	0.56 (0.54 – 0.59)	533.95	<0.001
Chronic Pulmonary Disease	0.84 (0.82 – 0.86)	192.39	<0.001	0.82 (0.80 – 0.85)	148.61	<0.001
Anemia	0.58 (0.56 – 0.60)	903.40	<0.001	0.71 (0.69 – 0.74)	282.89	<0.001
Electrolyte Imbalance	0.60 (0.58 – 0.61)	2109.36	<0.001	0.62 (0.60 – 0.63)	1493.67	<0.001
Sepsis	0.21 (0.19 – 0.23)	1497.25	<0.001	0.27 (0.25 – 0.30)	764.17	<0.001

## Discussion

The current study aimed to identify predictors of pacemaker implantation in patients hospitalized with syncope using a nationally representative sample (NIS) from 2016 to 2020 [8]. We found that older patients made up a larger proportion of the PPI group. This finding is consistent with well-known data linking advanced age to degeneration of the cardiac conduction system. Similarly, in a Spanish study of syncope patients with Holter devices, pacemaker implantation increased sharply with age, rising from 0.1% in patients younger than 50 to 9.3% in those aged 80 and older [10]. Additionally, our analysis showed that males constituted a greater proportion of the PPI group. This aligns with population-level data, including a large Australian study showing nearly two-fold higher age-adjusted PPI rates in men compared with women, a disparity that persisted after accounting for age and conduction disease. Notably, differences between genders remain evident even when analyses are restricted to patients hospitalized with arrhythmia, cardiomyopathy, or syncope. Across these settings, women with syncope were significantly less likely to receive PPI despite adjustment for age and comorbidities, raising concern for potential sex-based underutilization of device therapy [11].

Medicare is the primary insurance for individuals aged 65 years and above. It is therefore not surprising that among the PPI cohort, which largely comprises older-aged patients, most of them are Medicare beneficiaries. The association between PPI and lower income quartiles likely reflects the well-established bidirectional relationship between socioeconomic status and cardiovascular comorbidity, including conditions predisposing to conduction system disease. However, this finding may also indicate persistent under-referral of lower-income patients for specialist evaluation and device therapy despite substantial disease burden, a pattern that has been consistently observed across multiple cardiac device modalities in national datasets [12]. We also noted a higher proportion of White patients within the PPI cohort compared to non-PPI, as well as significant underrepresentation of Blacks, Hispanics, Asians, and Native American patients. To address this disparity in eligible patients receiving guideline-based pacemaker therapy, opportunities for intervention include improving referrals to specialists, addressing implicit bias held by healthcare providers, and increasing community outreach to improve equity across sex, race, and socioeconomic status [13,14].

An overwhelming majority of admissions in both the PPI and non-PPI cohorts were to Urban Teaching Hospitals, consistent with national hospitalization data indicating that most syncope admissions occur at academic medical centers. These centers are perceived to provide quality care, have increased availability of specialized cardiac electrophysiology services, and the ability to handle and process clinically complex patients with diagnostic heterogeneity often seen with syncope workups [15]. A higher proportion of syncope hospitalizations in both the PPI and non-PPI cohorts originated from the Midwest. This distribution parallels national emergency department data, in which Midwest residents account for approximately one-fifth of syncope-related visits, the second-highest regional contribution nationwide. The overrepresentation of Midwestern patients evaluated at metropolitan teaching hospitals likely reflects regional population density and the concentration of academic medical centers [16]. However, inferences drawn from our multivariable model regarding specific rural-versus-urban disparities should be interpreted with caution, as the necessary use of an 'unclassified' hospital reference group in our analysis precludes direct statistical comparison between defined hospital types.

The most discriminatory feature of our comorbidity analysis is likely the substantial difference in conduction abnormalities noted between our PPI and non-PPI cohorts. This was also noticed after adjusting for multiple confounding factors. This finding is also supported by a recent systematic review and meta-analysis that assessed implantable loop recorder (ILR)-monitored patients with syncope in 2024 and found that right bundle branch block and bifascicular block were the two strongest independent predictors of pacemaker requirement [17]. However, the sheer magnitude of this association in our analysis must be interpreted with caution. Due to the nature of administrative data, there is a high likelihood of indication bias; the ICD-10-CM code for a conduction disorder may frequently reflect the clinical justification for the pacemaker implantation itself, rather than a strictly independent, pre-existing comorbidity. Therefore, while clinically plausible, this specific adjusted odds ratio likely captures significant indication-related coding overlap.

The strong association between atrial fibrillation and increased odds of PPI aligns with shared structural remodeling that predisposes patients to tachycardia-bradycardia syndrome and subsequent sinoatrial node dysfunction [18]. Similarly, the independent association between obesity and increased odds of PPI is supported by evidence linking adiposity to structural alterations in the sinoatrial node and conduction pathways [19].

Conversely, several conditions were associated with significantly lower odds of pacemaker implantation, likely because they represent acute, reversible, or non-cardiac mechanisms of syncope. For example, syncope in the setting of sepsis or electrolyte imbalance is generally driven by acute hemodynamic or metabolic disturbances rather than intrinsic conduction system disease, which typically precludes the pursuit of long-term pacemaker therapy [20,21]. Similarly, in patients with a history of stroke, anemia, or chronic obstructive pulmonary disease, transient loss of consciousness is more frequently attributed to neurological, hypoxic, or hypoperfusion etiologies rather than bradyarrhythmias, resulting in lower rates of device implantation [22-24]. In the context of malignancy, reduced odds of PPI may additionally reflect provider reluctance to pursue invasive cardiac procedures in patients with limited life expectancy.

The shift of DM, CHF, CAD, and CKD to significant negative predictors after multivariate adjustment is a highly notable finding. Unadjusted, these comorbidities clustered with older age and conduction disease. However, once adjusting for the dominant effects of age (≥65 years) and baseline conduction disorders, these conditions inversely correlated with PPI. We hypothesize that in a fully adjusted model, these comorbidities serve as markers for alternative, non-bradycardic syncope mechanisms. For instance, DM and CKD may contribute to syncope predominantly through autonomic neuropathy and orthostatic hypotension [25-27]. Furthermore, conditions like CHF and CAD are clinically prioritized for implantable cardioverter-defibrillators to prevent sudden cardiac death, rather than permanent pacing for bradycardia [28,29].

The consistent pattern observed across all four shifted chronic illnesses, DM, CAD, CKD, and CHF, appears as markers of the sicker, older, and more conduction-disease burdened populations. However, after adjusting for age and conduction abnormalities (two of the greatest drivers of PPI indication), each comorbidity independently directs patients toward non-bradycardic causes of syncope that are inappropriate for pacing. Taken together, these findings suggest a hypothesized framework for risk stratification at the time of syncope hospitalization: patients with conduction disorders and age above 65 represent the highest-risk group for PPI; those with atrial fibrillation, obesity, or hypertension occupy an intermediate tier; and those presenting with DM, CHF, CKD, CAD, sepsis, or other reversible comorbidities as the dominant clinical picture are least likely to require permanent pacing. Clinicians should resist the assumption that the presence of these disease conditions increases the chances of a bradyarrhythmic etiology requiring pacing. Rather, after adjusting for confounders, these conditions may signal non-bradycardic mechanisms that require a more comprehensive evaluation, including tilt-table testing, prolonged ECG monitoring, autonomic function assessment, and structural imaging before pacemaker implantation is pursued.

Despite the advantage of the NIS dataset being the largest inpatient dataset in the US, it has some limitations. This database may underestimate the total number of PPIs as it does not capture outpatient procedures, and it may also have inappropriate coding. The available dataset is cross-sectional, and it fails to provide long-term outcomes. The quality of this dataset is also highly dependent on the experience of the coders. Furthermore, restricting our cohort to a primary diagnosis of syncope may exclude patients where syncope was coded secondarily to a broader cardiac admission. Additionally, because the NIS tracks hospital discharges rather than individual patients, a single patient admitted multiple times for syncope within the study period may appear as separate entries, introducing a degree of readmission overlap that we were unable to account for. The NIS also lacks the granular clinical data that are central to most pacing decisions: there is no electrocardiographic information, no PR interval, no specific bundle branch block morphology, no left ventricular ejection fraction, and no vital sign data. Importantly, we were also unable to account for the use of atrioventricular nodal blocking agents such as beta-blockers and calcium channel blockers, which are directly relevant to the evaluation of bradyarrhythmia-mediated syncope. Despite adjusting for a broad range of covariates, residual confounding from unmeasured clinical variables cannot be excluded. There is also the matter of indication bias, particularly regarding conduction disorders: in some cases, the ICD-10-CM code for a conduction disorder may reflect the pacing indication itself rather than a pre-existing comorbidity, which could contribute to the very high aOR observed for this predictor. The inclusion of 2020 data introduces a further consideration, as the COVID-19 pandemic significantly disrupted hospitalization patterns and elective procedure scheduling in ways our model cannot fully account for. Finally, while we identified predictors of PPI, the cross-sectional nature of the NIS introduces inherent temporal uncertainty; because we cannot definitively establish the sequence of events between the coded diagnoses and the procedure, our findings preclude any causal inference and should be interpreted accordingly.

## Conclusions

This study highlights the real-world experience of hospitalized syncope patients and the predictors of pacemaker implantation. Conduction abnormalities and age above 65 years were significant positive predictors of pacemaker implantation. DM, CHF, CKD, and CAD, which were previously positive predictors, became negative predictors of PPI after adjustment. This prompts further evaluation by clinicians for patients with these co-morbidities. Ultimately, recognizing these adjusted clinical and socioeconomic predictors can help refine risk stratification at the bedside and bring greater attention to the racial and socioeconomic disparities in access to guideline-directed device therapy that this study brings into focus.
